# ADAR1 promotes the epithelial-to-mesenchymal transition and stem-like cell phenotype of oral cancer by facilitating oncogenic microRNA maturation

**DOI:** 10.1186/s13046-019-1300-2

**Published:** 2019-07-17

**Authors:** Xue Liu, Yu Fu, Jiadong Huang, Meng Wu, Zhenxing Zhang, Rongyao Xu, Ping Zhang, Shouwei Zhao, Laikui Liu, Hongbing Jiang

**Affiliations:** 1 Jiangsu Key Laboratory of Oral Diseases, Nanjing Medical University, No. 136, Hanzhong Road, Nanjing, 210029 Jiangsu Province China; 2 Department of Oral and Maxillofacial Surgery, The Affiliated Stomatological Hospital of Nanjing Medical University, Nanjing, 210029 Jiangsu Province China; 3 Department of Oral Pathology, The Affiliated Stomatological Hospital of Nanjing Medical University, Nanjing, 210029 Jiangsu Province China

**Keywords:** ADAR1, Oral squamous cell carcinoma, Dicer, microRNA, Epithelial-mesenchymal transition, Cancer stem cells

## Abstract

**Background:**

Adenosine deaminases acting on RNA (ADARs) are involved in adenosine-to-inosine (A-to-I) editing and implicated in tumorigenesis and prognosis. Emerging evidence has indicated that ADAR1, an ADAR family member, participates in the regulation of various cancers; however, its biological function in oral squamous cell carcinoma (OSCC) remains unclear. This study aimed to determine the role of ADAR1 in OSCC progression.

**Methods:**

ADAR1 expression in both normal tissues and carcinoma tissues and in OSCC cell lines was examined by real-time PCR and western blotting. Gain-of-function and loss-of-function approaches were used to examine the effect of ADAR1 on the migration, invasion, epithelial-mesenchymal transition (EMT) and stemness of OSCC. Furthermore, the relationship between ADAR1 and Dicer was determined by co-immunoprecipitation, and the expression of OSCC-associated oncogenic miRNAs was evaluated by real-time PCR. For in vivo experiments, a xenograft model where OSCC cells stably expressing ADAR1 were implanted was used to investigate the effect of ADAR1 on tumor growth and progression, and the expression of ADAR1, PCNA, SOX2 and POU5F1 was further detected by immunohistochemistry. The impact of ADAR1 expression on the survival status of OSCC patients was determined by survival analysis.

**Results:**

ADAR1 was overexpressed in OSCC and significantly associated with poor patient survival. There was a positive correlation between ADAR1 and the migration, invasion, EMT and stemness of OSCC. Mechanistically, ADAR1 was physically associated with Dicer, and six OSCC-associated oncogenic miRNAs were increased in OSCC cells with ADAR1 overexpression. In the mouse xenograft model of OSCC, ADAR1 overexpression promoted tumor growth and progression. Moreover, ADAR1 was highly expressed in OSCC patients with low survival rates.

**Conclusions:**

Our findings demonstrated that ADAR1 may play a significant role in OSCC progression via combining with Dicer to regulate oncogenic miRNA maturation and further affect cell migration and invasion.

**Electronic supplementary material:**

The online version of this article (10.1186/s13046-019-1300-2) contains supplementary material, which is available to authorized users.

## Background

Oral squamous cell carcinoma (OSCC) is one of the most prevalent cancers and includes epithelial neoplasms of the oral cavity and oropharynx; OSCC is a serious and growing problem in many parts of the world [1, 2]. OSCC has a locoregional evolution, which is characterized by invasion of regional anatomical structures, and is prone to metastasis through lymphatic channels. Despite recent advancements in surgical techniques and other anticancer methods, the overall survival has improved little over the past few decades [3, 4]. A better understanding of the biology of OSCC is urgently needed for improving the poor outcome of this disease.

RNA editing, which was first discovered in trypanosome mitochondria, directly alters RNA sequences through post-transcriptional modifications [5, 6]. The most well known type of RNA editing is adenosine-to-inosine (A-to-I) editing, which is catalyzed by the enzyme family of adenosine deaminases acting on RNA (ADARs) targeting double-stranded RNA (dsRNA) [6–8]. Growing evidence supports the role of ADARs in various biological processes via RNA editing-independent mechanisms, including gene expression regulation [9], miRNA generation [3, 10, 11] and protein-protein complex formation [12, 13]. In humans, three ADAR proteins (ADAR1–3) have been identified, and ADAR1 has been verified to play important roles in a variety of diseases, such as cancer and infection [14, 15]. Recent results suggest that ADAR1 is upregulated in various solid cancers and associated with poor prognosis, but the biological significance of ADAR1 in OSCC remains largely unknown.

MicroRNAs (miRNAs) are approximately 22 nucleotides in length and post-transcriptionally regulate the expression of genes involved in multiple cellular functions [16, 17]. Some miRNAs (oncogenic miRNAs) play a vital role in tumor progression and therapeutic resistance [18]. Dicer, an RNase III gene family member, is an important component of the miRNA processing mechanism [19]. After primary transcripts of miRNA genes (pri-miRNAs) are cleaved into precursor transcripts of miRNA genes (pre-miRNAs) in the nucleus, pre-miRNAs are exported to the cytoplasm and cleaved into mature miRNAs by Dicer [3, 20]. ADARs are thought to participate in several steps during miRNA generation [21, 22]. Global suppression of miRNA synthesis in *ADAR1*-null mice led to an embryonic lethal phenotype [23]. Recent research has revealed that there is a robust interaction between Dicer and ADAR1 [3] and that ADAR1 increases the rate of miRNA processing by Dicer [6, 8]. In E11–12 embryos, upregulation of Dicer and ADAR1 p100 resulted in increased pre-miRNA processing capability [3].

In the present study, we found that ADAR1 was overexpressed in OSCC tissues and cell lines and was associated with poor prognosis. Additionally, we found that an abundance of ADAR1 enhanced the malignant phenotype, including stemness and EMT, of OSCC cell lines. Furthermore, we demonstrated that ADAR1 could interact with Dicer and promote the maturation of oncogenic miRNAs. Taken together, our findings showed that ADAR1 may be a key regulator of OSCC progression and a potential therapeutic target.

## Methods

### Patients and tissue specimens

Sixty-one OSCC tissue samples and matched noncancerous oral mucosa tissue samples (collected postoperatively from June 2014 to October 2015) used in this study were obtained from the Department of Oral Pathology, School of Stomatology, Nanjing Medical University. None of the patients had received radiotherapy or chemotherapy before their operation. Upon removal, the specimen was examined and divided into cancerous and normal tissues by pathology faculty. Each sample was snap-frozen in liquid nitrogen and stored at − 80 °C prior to RNA isolation. The tumor stage was determined according to the TNM classification system of the International Union Against Cancer. Tumor histological grade was determined according to Broder’s classification system. All surgical specimens were reviewed and analyzed after the study protocol was approved by the Ethics Committee of Nanjing Medical University. All procedures performed in studies involving human participants were in accordance with the 1964 Helsinki Declaration and its later amendments or comparable ethical standards. Informed consent for tissue donation for this research was obtained before specimen collection.

### Cell culture

Human OSCC cell lines (SCC9 and Cal27) were obtained from the American Type Culture Collection (ATCC). HOK cells were obtained from ScienCell, and HN4 and HN6 cells were from patients with head and neck squamous cell carcinoma (HNSCC). HN4, HN6, SCC9 and Cal27 cells were cultured in Dulbecco’s modified Eagle’s medium/F12 (DMEM/F12, Gibco, USA) containing 10% fetal calf serum (FBS, HyClone, USA), 100 U/ml penicillin and 100 μg/ml streptomycin (Invitrogen, USA). HOK cells were cultured in oral keratinocyte medium (OKM, ScienCell, USA) with 10% FBS, 100 U/ml penicillin and 100 μg/ml streptomycin. Cells were incubated in a humidified atmosphere at 37 °C in the presence of 5% CO_2_. All cell lines were passaged for fewer than 6 months.

### Real-time PCR

Total RNA was extracted from cells using TRIzol reagent (Invitrogen, San Diego, CA, USA). RNA was reverse transcribed into cDNA using a Primer-Script™ one-step RT-PCR kit (TaKaRa, Dalian, China). The cDNA template was amplified by real-time PCR using a SYBR® Pre-mix Dimmer Eraser kit (TaKaRa, Dalian, China). *GAPDH* and *U6* were used as internal controls, and mRNA and miRNA values were normalized to *GAPDH* and *U6*, respectively. The primers used were as follows: *ADAR1*, F: 5′ - TGCTGCTGAATTCAAGTTGG - 3′; R: 5′ - TCGTTCTCCCCAATCAAGAC- 3′, *ADAR1-p110*, F: 5′ -GGCAGCCTCCGGGTG- 3′; R: 5′ -CTGTCTGTGCTCATAGCCTTGA- 3′, *ADAR1-p150*, F: 5′ -CCACCTCCAGTGCGGAGTAGCG- 3′; R: 5′ -TGCCCCTTGAGAAATTCTATTTGC- 3′, *Dicer*, F: 5′ -TCCACGAGTCACAATCAACACGG- 3′; R: 5′ -GGGTTCTGCATTTAGGAGCTAGATGAG- 3′, *GAPDH*, F: 5′ - CCGGGAAACTGTGGCGTGATGG - 3′; R: 5′ - AGGTGGAGGAGTGGGTGTCGCTGTT- 3′. Real-time PCR was performed using an ABI7900 system (Applied Biosystems, CA). The relative expression fold change of mRNAs was calculated by the 2^-ΔΔCt^ method. All miRNA and *U6* primers were purchased from Genecoponeia, Guangzhou, China.

### Western blot analysis

Harvested cells were lysed on ice for 30 min in RIPA Lysis Buffer (Beyotime Biotechnology, Shanghai, China) containing 100 mM PMSF (Beyotime Biotechnology) and centrifuged at 12000×g for 10 min to collect total protein samples. To isolate the cytoplasmic components from the nuclear components, the cells were mechanically homogenized and treated with a nuclear protein extraction kit (Beyotime Biotechnology). The protein lysate supernatants were mixed with loading buffer, separated on a 10% SDS-polyacrylamide gel and transferred to an Immobilon-PVDF membrane (Millipore Corporation, Billerica, MA, USA). The membranes were blocked with 5% non-fat milk at 22 °C and incubated with primary antibodies at 4 °C overnight. Detailed information on the primary antibodies is provided in Additional file 5: Table S1. The membranes were incubated with secondary peroxidase-conjugated antibodies. Finally, the protein bands on the membranes were visualized using chemiluminescence reagents (WBKLS0100; Millipore Corporation, Billerica, MA, USA) according to the manufacturer’s instructions.

### RNA interference

Small interfering RNA (siRNA) targeting ADAR1 and scrambled siRNA were designed and synthesized by GenePharma (Shanghai, China). Three sequences of siRNAs targeting *ADAR1-p110* were used: siRNA1, 5′ - CCUUCUACAGUCAUGGCUUTT - 3′, 3′ - AAGCCAUGACUGUAGAAGGTT − 5′; siRNA2, 5′ - CCACUAUUCCACAGAGAAATT -3′, 3′ - UUUCUCUGUGGAAUAGUGGTT − 5′; siRNA3, 5′ - CCAUGAACCCAAGUUCCAATT -3′, 3′ - UUGGAACUUGGGUUCAUGGTT − 5′. The siRNA sequence used for targeting *Dicer* was 5′ - GCCAAGGAAAUCAGCUAAATT -3′, 3′ - UUUAGCUGAUUUCCUUGGCTT − 5′. According to the literature [24], the siRNA sequence used for targeting *ADAR1-p150* was 5′ - GCCUCGCGGGCGCAAUGAATT -3′, 3′ - UUCAUUGCGCCCGCGAGGCAT − 5′. All of these siRNA duplexes (final concentration 50 nM) were transfected into cells using Lipofectamine 2000 (Invitrogen, San Diego, CA, USA) according to the manufacturer’s instructions. Knockdown efficiency was determined after 48 h of culture.

### Lentiviral vector construction and transfection

The *ADAR1 p110* mRNA sequence (GenBank Accession NM_001025107.2) was synthesized and subcloned into the LV5 (EF-1aF/GFP &Puro) vector (LV-*ADAR1*), and the empty LV5 vector with the GFP gene (LV-GFP) served as a negative control (GenPharma, Shanghai, China). Cells were infected with LV-*ADAR1* or LV-GFP and then cultured with DMEM containing 10% FBS in the presence of 0.5 μg/ml puromycin for 7 days. Stable clones of the cells were selected and used in the following experiments.

### Wound healing assay and invasion assay

For wound healing assays, HN4 and Cal27 cells were seeded in a 6-well plate and then cultured in growth medium until they reached 80% confluency. The monolayer was then disrupted with a 1.2-mm cell scraper. Next, we used PBS to wash away the non-adherent cells and debris, and the cells were incubated with serum-free medium for 18 h. Lesion areas were imaged at 0 and 18 h under a phase-contrast microscope. The invasion assay was performed using Matrigel-coated transwell inserts. Briefly, 5 × 10^4^ cells in 250 μl of serum-free medium were seeded into the upper chamber, and 750 μl of medium was added to the lower chamber. After incubation for 24 h, the chambers were first fixed in 4% paraformaldehyde for 30 min and then stained with a 0.05% crystal violet solution for 15 min. The numbers of cells at 100× magnification were counted using a positive microscope. Three random fields were recorded for each well.

### Immunofluorescence

Cells were incubated for 24 h to reach approximately 60% confluence and then fixed with 4% paraformaldehyde and permeabilized in 0.5% Triton X100 for 10 min. Next, antigens were blocked for 1 h with 1% BSA, and the cells were incubated with primary antibodies at 4 °C overnight. The cells were washed with PBS, incubated for 1 h with secondary antibody and stained with DAPI. Images were acquired with a Laser scanning Confocal Microscopy and statistical analysis was performed by Image J software.

### Subcutaneous nude mouse xenografts

Ten 4-week-old male nude mice (Institute of Zoology, China Academy of Sciences) were divided randomly into 2 groups (5 in each group). One million Cal27/Vector or Cal27/LV5-p110 cells in 100 μl of PBS were inoculated subcutaneously. Tumor nodules were measured every 7 days and calculated by the following formula: V = (Width^2^ × Length)/2. Xenografts were collected at the 5 th week for immunohistochemistry staining.

### 3D Colony formation assay

Briefly, 1000 cells per well were cultured in ultra-low attachment culture plastic ware (Corning Incorporated, catalog number: 3473 24-well plate, USA) with DMEM containing 10% Matrigel, 10 ng/ml EGF, 20 ng/ml FGF, 1× B27, and 5 μg/ml insulin. After 7 to 14 days, the cell colonies (diameter > 50 μm) were counted microscopically. Five fields per well were used to calculate the average value.

### Co-immunoprecipitation

Cells were harvested and lysed using sonication in cell lysis buffer (50 mM Tris-HCl, pH 7.5, 130 mM NaCl, 1% Nonidet P-40, 0.5% sodium deoxycholate and 1% protease inhibitor cocktail). Cell lysates were centrifuged at 5000 R/M, and the supernatants were incubated with the indicated antibodies and Protein A/G PLUS-Agarose beads (sc-2003, Santa Cruz, USA) at 4 °C overnight. The beads were washed three times with cell lysis buffer, and the precipitated proteins were further analyzed. For western blot details, please see the related assay section above.

### Immunohistochemistry

Paraffin-embedded surgical tissue specimens were from 108 patients with OSCC who underwent surgery at the Affiliated Hospital of Stomatology, Nanjing Medical University. The specimens were sectioned at 5-μm thickness, and the sections were baked at 37 °C overnight. The sections were deparaffinized with xylene and hydrated with graded alcohol. Antigen retrieval was conducted by heating for 15 min in a pressure cooker with citrate buffer. Endogenous peroxidase was blocked by incubation with 3% H_2_O_2_ plus 1% Triton X100 in paraformaldehyde for 20 min at room temperature. The sections were then incubated overnight with primary antibodies for ADAR1 (1:50 dilution, Abcam, USA), PCNA (1:250 dilution, Abcam, USA), SOX2 (1:300 dilution, Cell Signaling Technology, USA) and POU5F1 (1:500 dilution, Proteintech, USA). Specimens were reacted with a secondary peroxidase-conjugated antibody, and the reaction products were visualized with a 3,3 -diaminobenzidine (DAB) solution. The samples were scored independently by two researchers who were blinded to the clinicopathological data at 200× magnification under a light microscope. The evaluation was based on the staining intensity and extent of staining. Staining intensity was scored as 0 (negative), 1 (weak) and 2 (strong). Staining extent was scored as 0 (0%), 1 (1–50%) and 2 (51–100%), depending on the percentage of positively stained cells. Positive staining was determined by the following formula: overall score = percentage score × intensity score. An overall score of ≤1 was defined as low expression, and an overall score of ≥2 was defined as high expression.

### Flow cytometric analysis

Cells were seeded at a density of 1 × 10^6^ cells/well in six-well plates. After 24 h, the cells were washed with PBS, fixed in ice-cold 70% ethanol for 1 h and then treated with 100 μL of 50 mg/L propidium iodide for 30 min at 4 °C in the dark. The cell cycle profiles were assayed using an Elite ESP flow cytometer at 488 nm, and the data were analyzed with the CELL Quest software (BD Biosciences, San Jose, CA, USA).

### Cell growth assessment

Cell growth was assessed by the Cell counting Kit-8 (CCK-8; Medchem Express, New Jersey, USA) according to manufacturer instructions at 12, 24, 36, 48, 72 h. Cells were cultivated into 96-well culture plates with a density of 6 × 10^3^ per well. Briefly, absorbance values were determined by the microplate reader at a wavelength of 450 nm. Each experiment was repeated three times and data are presented as the mean.

### Statistical analysis

All statistical analyses were performed using SPSS 17.0 and GraphPad Prism 7 software. The expression differences between high/low T category, N category, histological grades, high/low stages, cell lines, expression changes after transfection, protein expression, immunohistochemistry, cell migration and cell invasion assays were analyzed using the independent samples t-test. A chi-square test was used to evaluate the association between the expression of ADAR1 and the clinicopathological features and prognostic factors of OSCC. Kaplan-Meier analysis was used to assess the survival rate curves, and statistical significance was determined using the log-rank test. Univariate and multivariate Cox regression models were performed for the prognostic analyses. All data are presented as the mean ± standard error. A two-sided *p* value of less than 0.05 was considered statistically significant.

## Results

### ADAR1 is markedly upregulated in OSCC tissues and cell lines

**Fig. 1:**
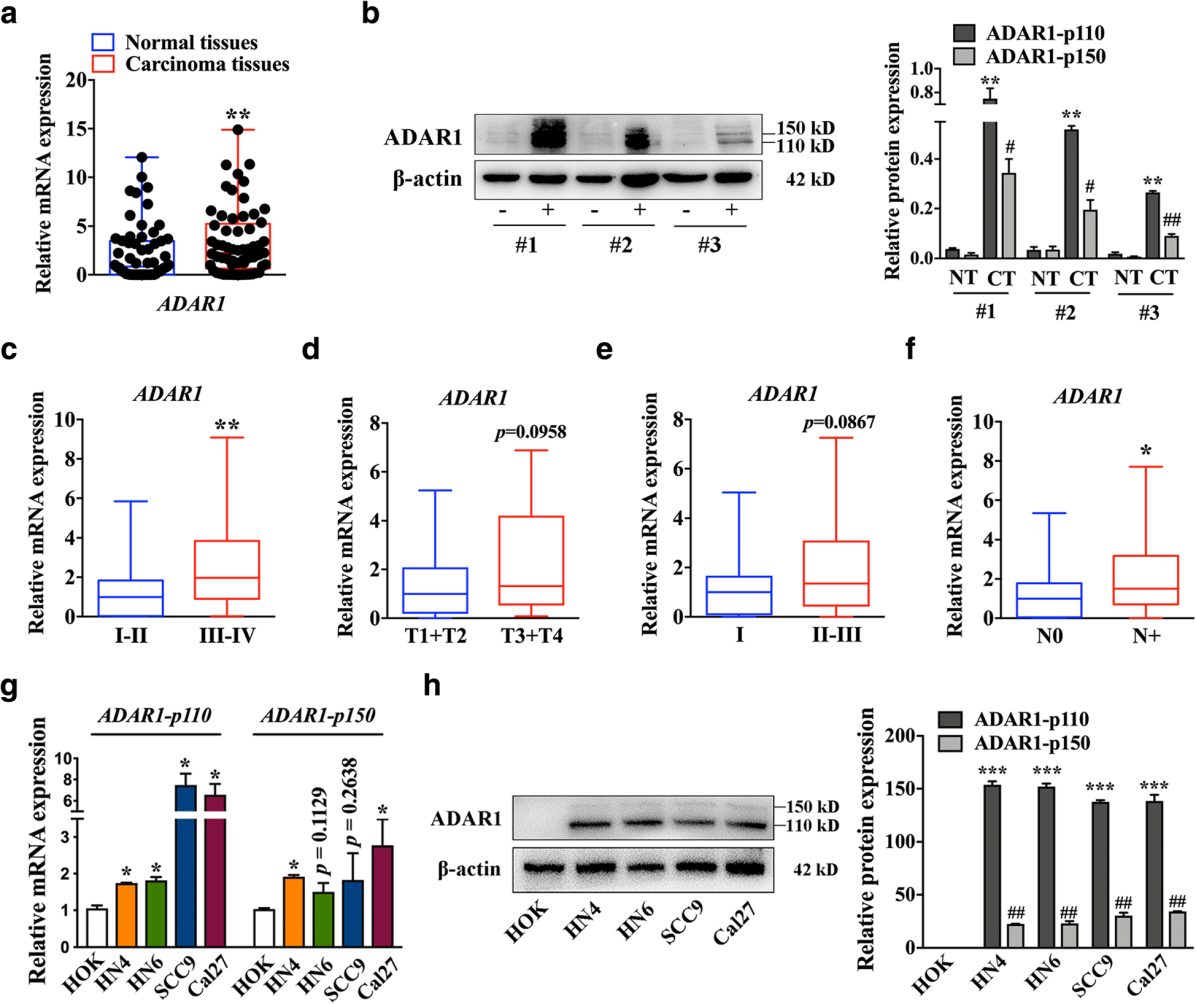
ADAR1 expression in OSCC and its clinical significance. **a** Differences in the expression of *ADAR1* between OSCC tissue and matched non-tumor oral mucosa. The expression of *ADAR1* was normalized to that of *GAPDH*. Significant differences between samples were analyzed with the paired Wilcoxon test and paired t test (*n* = 61, *p* < 0.01). **b** ADAR1 protein expression in tissue from three random patients. -, non-tumor oral mucosa; +, OSCC tissue. **c** Relationship between ADAR1 expression and TNM stage (*p* < 0.01). **d** Relationship between ADAR1 expression and primary tumor growth (*p* = 0.0958). **e** Relationship between ADAR1 expression and pathological grade (*p* = 0.0867). **f** Relationship between ADAR1 expression and lymph node metastasis (*p* = 0.05). **g** mRNA levels of *ADAR1-p110* and *ADAR1-p150* in HOK and OSCC cell lines. **h** ADAR1 protein expression in HOK and OSCC cell lines. The data were summarized from at least three independent experiments. *p < 0.05; **p < 0.01; ***p < 0.00; *#p* < 0.05; ##*p* < 0.01

To determine the expression of ADAR1 in OSCC patients, the epicenter of the tumor tissues and matched distal normal tissues from 61 patients were examined by real-time PCR. The results revealed that tumor tissues had markedly higher levels of *ADAR1* than matched distal normal tissues (Fig. 1a). Meanwhile, the expression of *ADAR1-p110* and *ADAR1-p150* in OSCC patients was detected and the result showed both *ADAR1-p110* and *ADAR1-p150* were enhanced expression in OSCC tissues (Additional file 1: Figure S1). The ADAR1 protein was similarly overexpressed in OSCC tissues that were harvested from 3 random patients (Fig. 1b). To better understand the underlying biological significance of ADAR1, we assessed the relationship between ADAR1 expression and the clinicopathological profiles of OSCC patients. Details of the clinical features are shown in Additional file 6: Table S2. ADAR1 expression was positively correlated with TNM staging (Fig. 1c) and lymph node metastasis (Fig. 1f). Although the data for different T categories and histology grades were not significantly different (Fig. 1d, e), ADAR1 tended to be higher in patients with a higher T stage and poor differentiation. We then focused on the expression of ADAR1 in OSCC cell lines. Consistent with the tissue results, ADAR1 expression was higher in OSCC cell lines than in human oral keratinocyte (HOK) cells (Fig. 1g, h). Together, these results revealed that ADAR1 was overexpressed in both OSCC tissues and cell lines and suggested that ADAR1 might play an important role in OSCC.

### ADAR1 promotes migration, invasion, and proliferation in OSCC

**Fig. 2:**
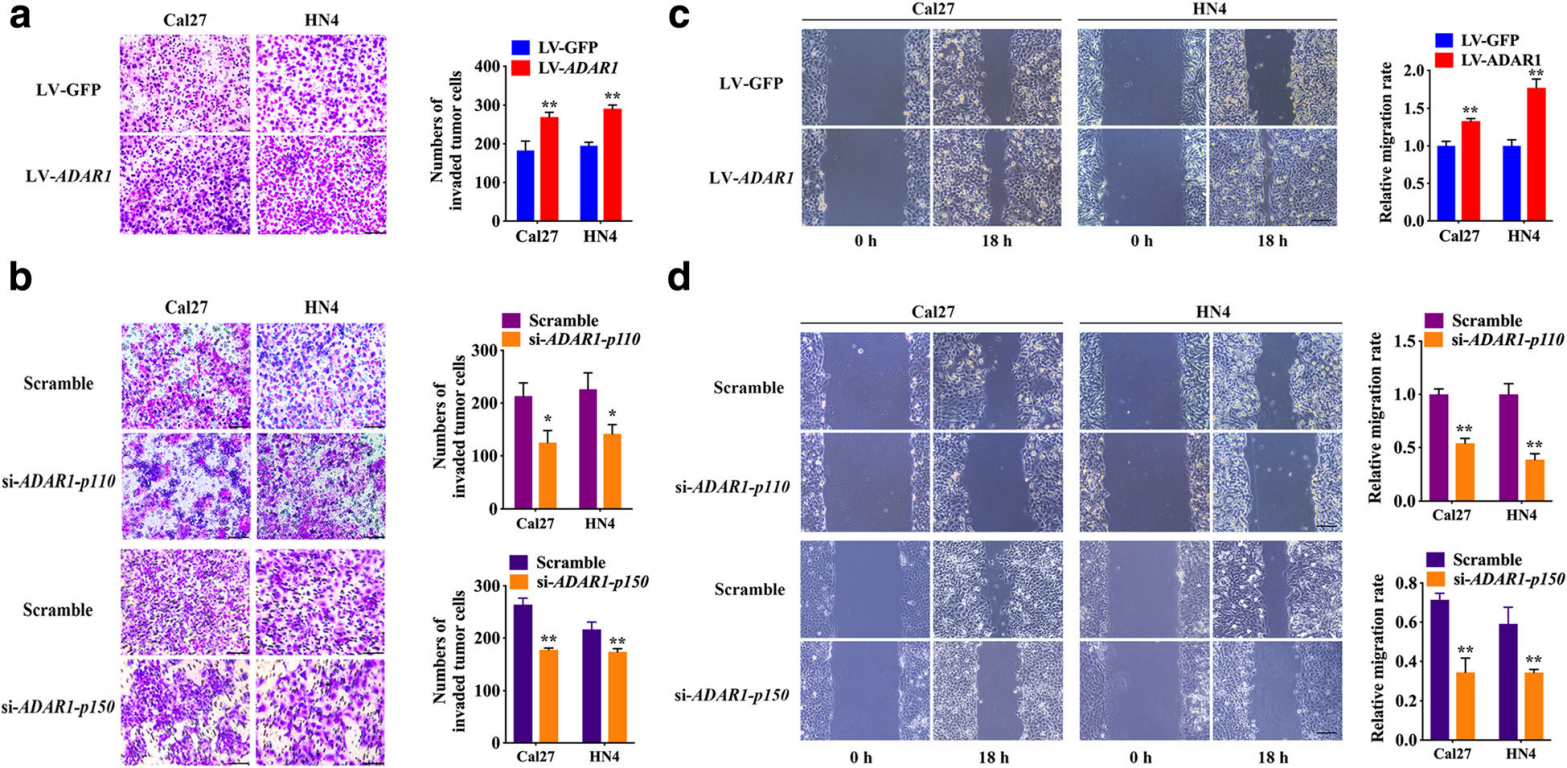
Effect of ADAR1 on the invasion, migration of OSCC cell lines. **a** Representative images of the invasion assay using OSCC cell lines transfected with the ADAR1 overexpression vector (LV-*ADAR1*) and the respective vector control (LV-GFP). **b** Representative image of the invasion assay using OSCC cell lines transfected with *ADAR1-p110*, *ADAR1-p150* siRNA (si-*ADAR1-p110,* si-*ADAR1-p150*) and the respective scrambled vector control (Scrambled). **c** A scratch-wound assay was used to study the migration ability of LV-*ADAR1* and LV-GFP cells over a period of 18 h. **d** Migration assay of both si-*ADAR1-p110* and si-*ADAR1-p150* groups during a period of 18 h

To further learn the role of ADAR1 in OSCC, we chose HN4 and Cal27 as our model cell lines. A Conclusion Lentiviral vector carrying *ADAR1 p110* gene was successfully constructed and used to induce high expression in both OSCC cell lines, and ADAR1 expression was decreased by siRNA transfection (Additional file 2: Figure S2a-f). In the invasion study, obviously more HN4 and Cal27 cells with ADAR1 overexpression (LV-*ADAR1*) penetrated the membrane of the chambers than control cells (LV-GFP) (Fig. 2a). Both HN4 and Cal27 cells with either *ADAR1-p110* knockdown (si-*ADAR1-p110*) or *ADAR1-p150* knockdown (si-*ADAR1-p150*) showed weaker invasion abilities than control cells (scrambled) (Fig. 2b). A wound-healing assay was then performed to detect the role of ADAR1 in the progression of OSCC. In the wound healing experiment, the LV-*ADAR1* group was significantly faster than the LV-GFP group (Fig. 2c). Conversely, both si-*ADAR1-p110* and si-*ADAR1-p150* attenuated the migration of HN4 and Cal27 cells (Fig. 2d). Furthermore, the flow cytometry results revealed that upregulation of ADAR1 promoted cell proliferation by increasing the proportion of OSCC cells in S-phase (Additional file 2: Figure S2g). The CCK8 assay results also implied that LV-*ADAR1* group exhibited enhanced cell proliferation ability comparing with LV-GFP at 72 h (Additional file 2: Figure S2h, i). These results may imply that ADAR1 promoted the migration, invasion and proliferation of OSCC cell lines.

### ADAR1 participates in the process of TGF-β-induced EMT in OSCC

**Fig. 3:**
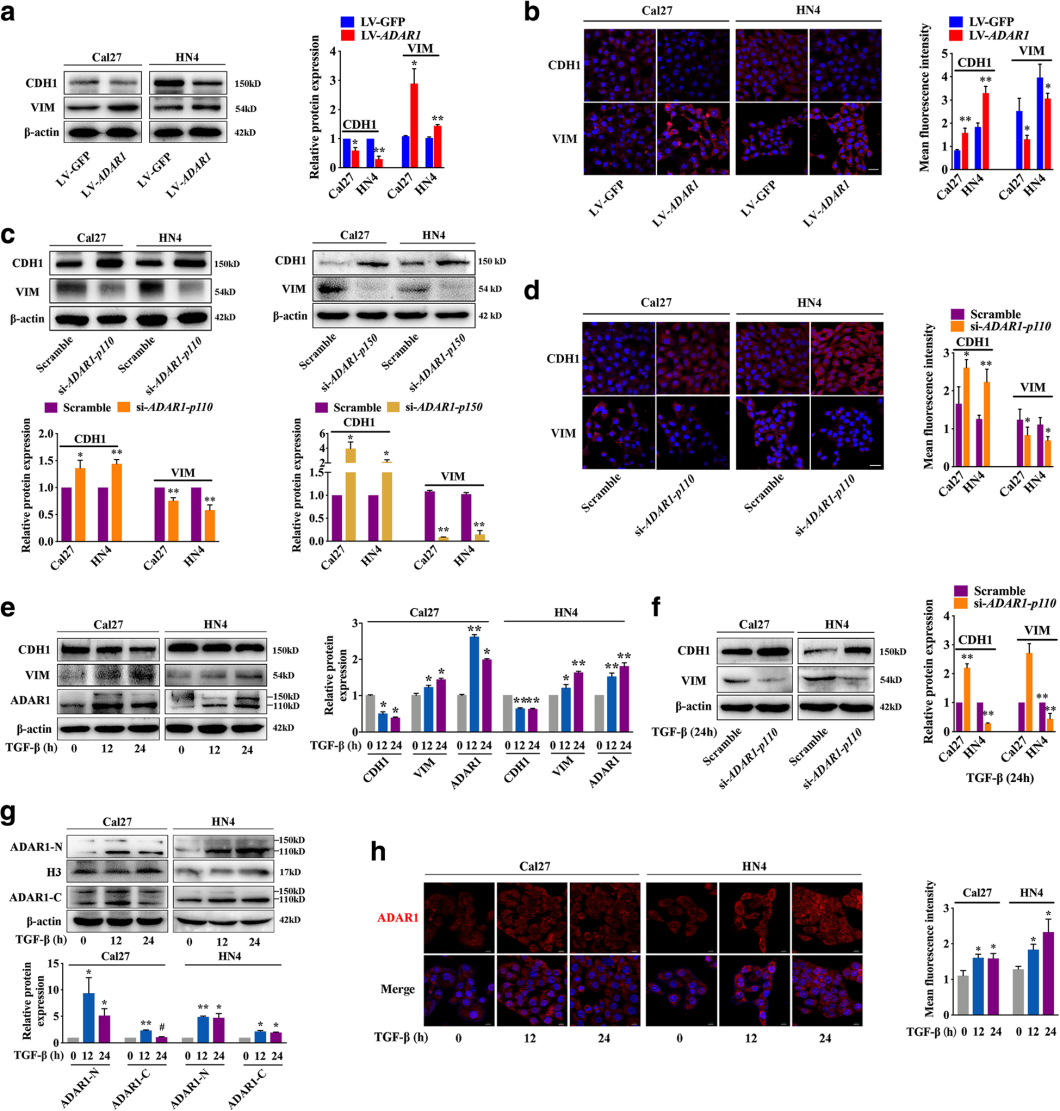
Effect of ADAR1 on EMT of OSCC cell lines. **a** Western blot analysis of CDH1 and VIM in the LV-*ADAR1* and LV-GFP groups. **b** CDH1 and VIM protein expression was detected by immunofluorescence analysis. **c** Western blot analysis of CDH1 and VIM in both si-*ADAR1-p110* and si-*ADAR1-p150* groups. **d** Representative image of immunofluorescence indicating that *ADAR1-p110* knockdown attenuated EMT in OSCC cell lines. **e** Western blot analysis of CDH1, VIM and ADAR1 in OSCC cell lines treated with TGF-β for 0, 12, and 24 h. **f** Western blot analysis of CDH1 and VIM in the Scramble and si-*ADAR1-p110* groups with 24 h TGF-β treatment. **g** The nuclear (ADAR1-N) and cytoplasm (ADAR1-C) expression levels of ADAR1 were measured in OSCC cell lines treated with TGF-β for 0, 12, and 24 h by western blot analysis. **h** Representative image of immunofluorescence showing ADAR1 expression in the process of TGF-β treatment. Scale bars: 20 μm. The data from at least three independent experiments were expressed as the mean ± SEM. *p < 0.05; **p < 0.01; #*p* > 0.05

Previous studies have confirmed that EMT may play a fatal role in OSCC [25]. To examine whether ADAR1 enhanced the EMT of OSCC, the protein expression of CDH1 and VIM was examined by western blot. Both Cal27 and HN4 cells transfected with LV-*ADAR1* had higher expression of VIM but lower expression of CDH1 than cells transfected with LV-GFP (Fig. 3a). Conversely, both si-*ADAR1-p110* and si-*ADAR1-p150* group showed less mesenchymal characteristics than the scrambled cells (Fig. 3c). Similar results were observed by immunofluorescence (Fig. 3b, d), thus validating that ADAR1 could promote EMT in OSCC. Cal27 and HN4 cells were exposed to 10 ng/ml TGF-β [26], which induced EMT, and the protein expression of VIM, CDH1 and ADAR1 was examined at 12 h and 24 h (Fig. 3e). Both Cal27 and HN4 cells showed mesenchymal characteristics at 12 h following increased ADAR1 expression. Higher CDH1 expression but lower VIM expression were detected in Cal27 and HN4 cells transfected with si-*ADAR1-p110* under 24 h TGF-β treatment (Fig. 3f). Cal27 and HN4 cells with *ADAR1-p110* overexpression were transfected by *ADAR1-p150* siRNA and reduced VIM expression and enhanced CDH1 expression were detected by western blot assay (Additional file 3: Figure S3). As shown in Fig. 3g, ADAR1 expression was upregulated at 12 h. Similarly, immunofluorescence also revealed that ADAR1 expression was significantly increased in both the nucleus and cytoplasm (Fig. 3h) upon TGF-β treatment. Collectively, we suggest that ADAR1 is involved in the EMT process of OSCC.

### ADAR1 maintains the stemness of OSCC in vitro

**Fig. 4:**
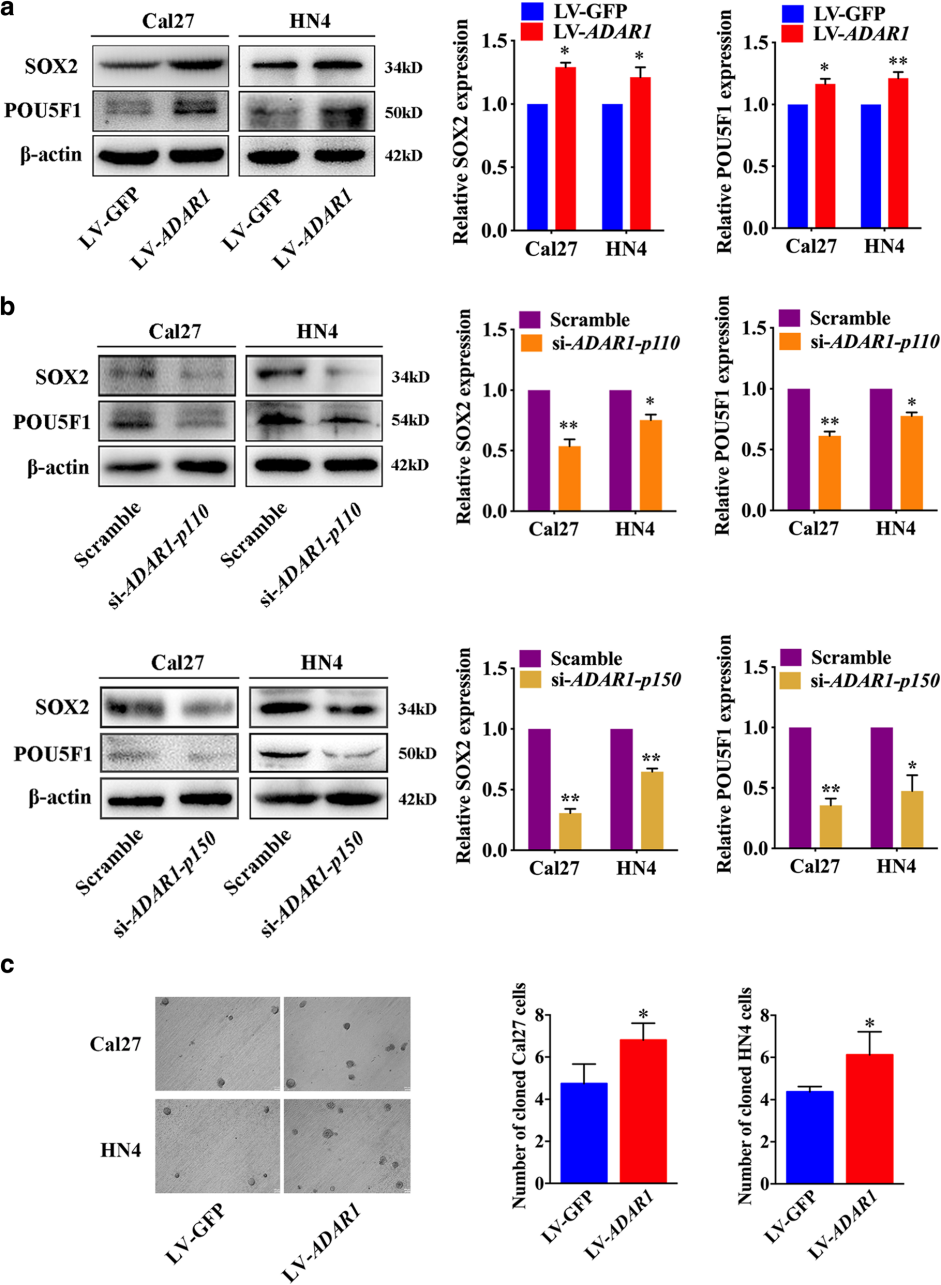
ADAR1 promoted the stemness of OSCC cell lines in vitro. **a** Western blot analysis of SOX2 and POU5F1 in LV-*ADAR1* and LV-GFP groups. **b** Western blot analysis of SOX2 and POU5F1 in si-*ADAR1-p110*, si-*ADAR1-p150* and scrambled groups. **c** Representative image of suspended cell culture indicating the colony formation ability of the LV-*ADAR1* and LV-GFP groups. The data were expressed as the mean ± SEM (*n* ≥ 3). *p < 0.05; **p < 0.01

We then determined whether ADAR1 could promote the stemness of OSCC cell lines. The protein expression of SOX2 and POU5F1 was examined by western blot. The LV-*ADAR1* group exhibited higher expression of SOX2 and POU5F1 than the LV-GFP group (Fig. 4a), whereas si-*ADAR1-p110* and si-*ADAR1-p150* groups attenuated the stemness of OSCC cell lines (Fig. 4b). To validate our observation, a suspended cell culture assay revealed more suspended cells in the LV-*ADAR1* group than in the LV-GFP group (Fig. 4c). However, the same assay using *ADAR1-p110* and *ADAR1-p150* knockdown cells was unsuccessful because of the extremely small number of suspended cells. These findings demonstrated that ADAR1 could maintain and promote the stemness of OSCC.

### ADAR1 physically interacts with Dicer and is involved in the maturation of oncogenic miRNAs

**Fig. 5:**
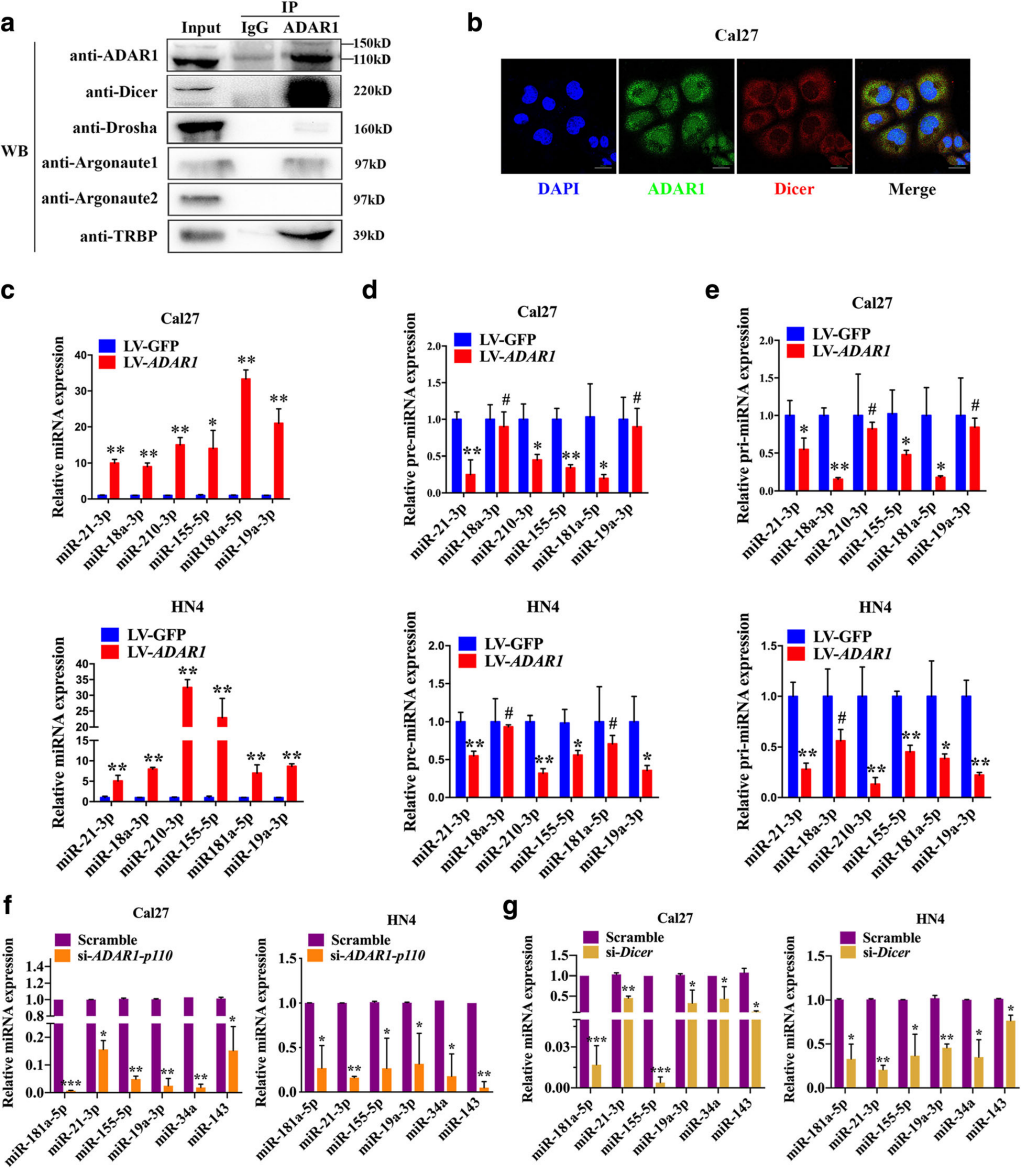
ADAR1 could bind to Dicer and promote the maturation of oncogenic miRNAs. **a** Representative image of Co-IP. Cell lysates were incubated with normal rabbit IgG, anti-ADAR1, anti-Dicer, anti-Drosha, anti-Argonaute1, anti-Argonaute2, and anti-TRBP antibodies. **b** Representative image of immunofluorescence indicated that ADAR1 and Dicer were co-located in Cal27 cells. Scale bar: 20 μm. **c** *ADAR1* overexpression upregulated oncogenic miRNAs. **d** *ADAR1* overexpression decreased the precursor levels of six oncogenic miRNAs. **e** *ADAR1* overexpression decreased the primary levels of six oncogenic miRNAs. **f** *ADAR1-p110* knockdown reduced oncogenic miRNAs and tumor suppressor miRNAs expression. **g** *Dicer* knockdown inhibited oncogenic miRNAs and tumor suppressor miRNAs expression. The data were expressed as the mean ± SEM (n ≥ 3). *p < 0.05; **p < 0.01; ***p < 0.00; #*p* > 0.05

ADAR1 p110 has been confirmed to increase the rate of miRNA processing by Dicer [3], so co-immunoprecipitation was performed to further verify the interaction of ADAR1 and Dicer in Cal27 cells (Fig. 5a). The results suggested that ADAR1 could mainly interact with Dicer comparing with Drosha, Argonaute1, Argonaute2 and TRBP. Immunofluorescence was also performed to validate our conclusion and showed that ADAR1 and Dicer were co-localized in the cytoplasm (Fig. 5b). The Pearson’s coefficients of co-localization assay was 0.45 ± 0.11. MiR-21–3p, miR-18a-3p, miR-210-3p, miR-155-5p, miR-181a-5p and miR-19a-3p were found to be oncogenic miRNAs according to supporting experimental data that showed worse tumor prognosis for HNSCC [27–31]. Increased expression in Cal27 cells treated with 10 ng/ml TGF-β for 24 h implied that these miRNAs may be involved in the EMT process (Additional file 4: Figure S4). We further examined whether ADAR1 could promote the maturation of these oncogenic miRNAs. Six mature oncogenic miRNAs were remarkably increased in the LV-*ADAR1* group (Fig. 5c). However, the precursors of these six oncogenic miRNAs were significantly lower in the LV-*ADAR1* group than in the LV-GFP group (Fig. 5d, e). Conversely, both oncogenic miRNAs and tumor suppressor miRNAs were reduced expression in either si-*ADAR1-p110* or si-*Dicer* group (Fig. 5f, g). These findings implied that ADAR1 could physically interact with Dicer and promote the formation of oncogenic miRNAs in OSCC.

### ADAR1 promotes tumor growth in vivo

**Fig. 6:**
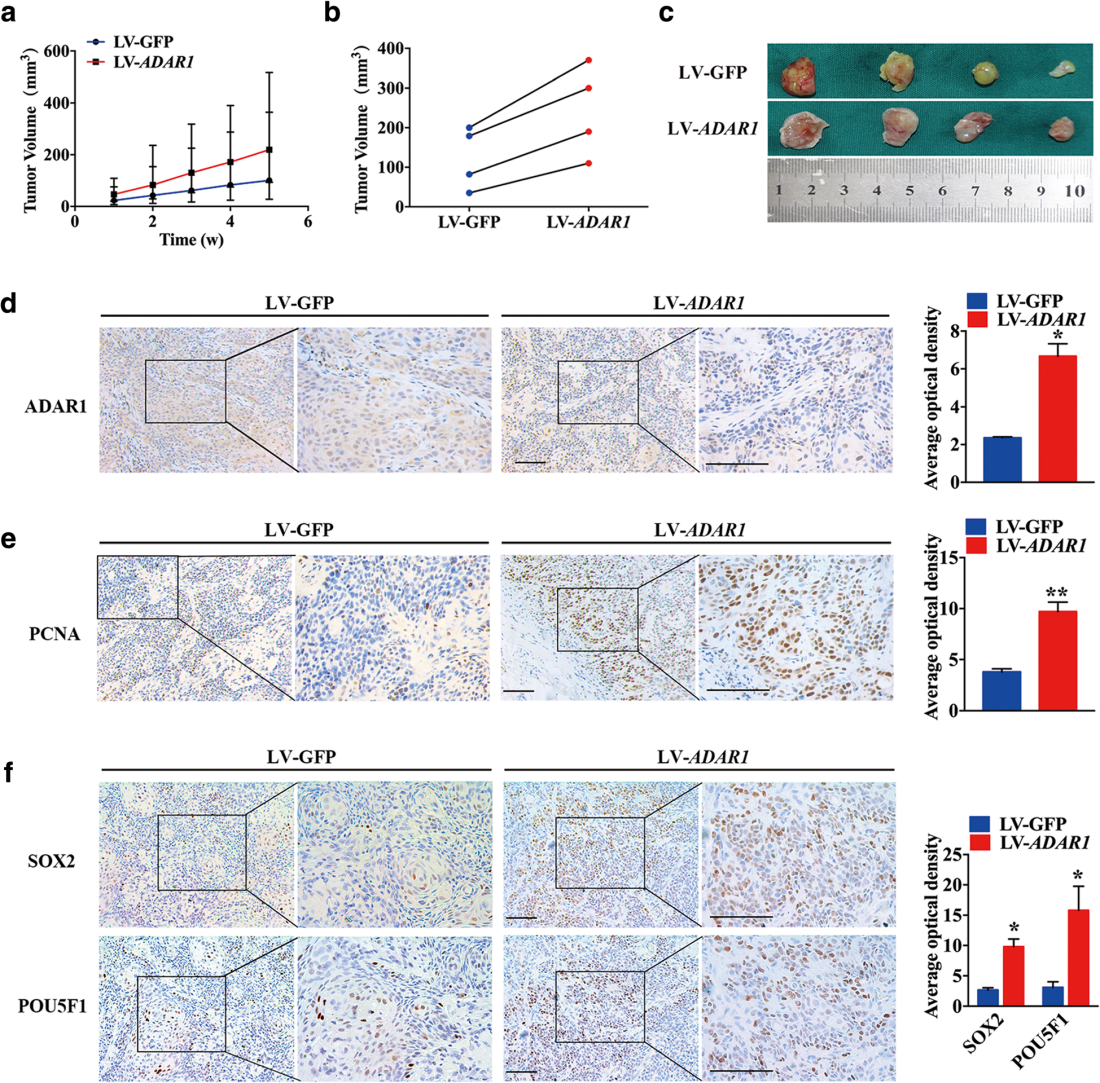
ADAR1 promoted tumor growth and stemness in the nude mouse xenograft model. **a** Image representing the volume of tumors after injection in ten nude mice (*p* < 0.05). Significant differences were analyzed using an independent t test. **b** Image representing the differences in the volume of tumors from nude mice injected with either LV-GFP or LV-*ADAR1* cells. **c** Image of excised tumors from nude mice with LV-*ADAR1* or LV-GFP cell injection. **d** and **e** ADAR1 and PCNA expression levels were examined by IHC. **f** SOX2 and POU5F1 expression levels were examined by IHC. Scale bars: 100 μm and 200 μm

Given the promising results found in vitro, we attempted to assess the effect of ADAR1 in murine models. The growth rate of xenografts with LV-*ADAR1* cells was remarkably faster than that of xenografts with LV-GFP cells (Fig. 6a), and the paired tumor weights of the xenografts were also considerable (Fig. 6b). Tumors from the LV-*ADAR1* group were larger than those from the LV-GFP group (Fig. 6c). ADAR1 expression in xenografts was detected by IHC (Fig. 6d) and was significantly higher in the LV-*ADAR1* group than in the LV-GFP group. Notably, the expression of PCNA was also positively increased in the LV-*ADAR1* group (Fig. 6e), indicating that ADAR1 may promote OSCC proliferation in vivo. As shown in Fig. 6f, the stemness markers were augmented correspondingly in the LV-*ADAR1* group according to an immunohistochemistry analysis of SOX2 and POU5F1.

### ADAR1 is overexpressed in OSCC patients and associated with poor prognosis

**Fig. 7:**
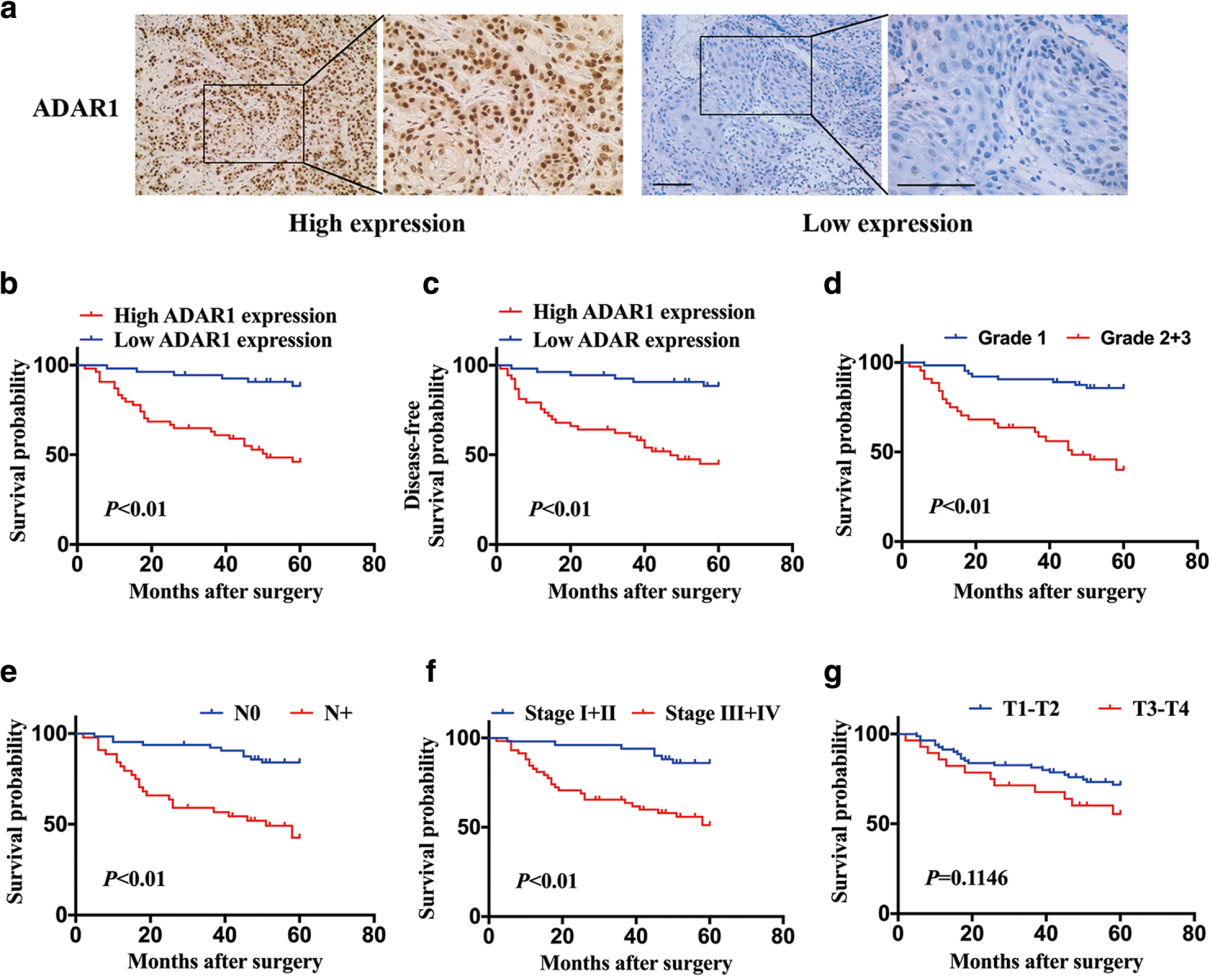
The prognosis of patients correlated with ADAR1 expression and clinical features. **a** Representative image indicating high expression and low expression of ADAR1 in OSCC tissues. **b** Overall survival of OSCC patients with high and low expression levels. **c** Disease-free survival of OSCC patients with high and low expression levels. **d** Overall survival of OSCC patients with different histopathological grades. **e** Overall survival of OSCC patients with different lymph node metastasis levels. **f** Overall survival of OSCC patients with different TNM stages. **g** Overall survival of OSCC patients with different primary tumor sizes

Paraffin sections from 108 patients were examined by IHC to assess the expression of ADAR1. ADAR1 protein expression in the OSCC samples was categorized as high or low (Fig. 7a). The relationship between ADAR1 and the clinicopathological features is shown in Table 1. The abundance of ADAR1 was positively associated with primary tumor size, lymph node metastasis and TNM stage. However, there was no remarkable correlation between ADAR1 and other clinicopathological features, such as gender, age or histology grade of the patient.

**Table 1:** The association between ADAR1 and clinicopathological features of OSCC (*n* = 108)

Clinicopathological features	Total (n)	ADAR1		χ^2^	*P*
		Low (n)	High (n)		
Gender					
Male	64	34	30	0.6136	0.4334
Female	44	20	24		
Age					
< 60	45	20	25	0.9524	0.3291
≥ 60	63	34	29		
T category					
T1 + T2	66	43	23	15.58	< 0.0001
T3 + T4	42	11	31		
N category					
N0	64	45	19	25.93	< 0.0001
N+	44	9	35		
Histology grade					
Grade 1	80	37	43	0.7249	0.3945
Grade 2 + 3	28	17	11		
TNM stage					
I + II	50	32	18	7.299	0.0069
III + IV	58	22	36		

*ADAR1* Adenosine deaminases acting on RNA, *OSCC* Oral squamous cell carcinoma, *T* Tumor size, *N* Lymph node, *TNM* Tumor-node-metastases, *P P* values of two-sided χ2 test; Both values signify *P*-value < 0.05

Further indicating the impact of ADAR1 expression on the survival status of OSCC patients, ADAR1 upregulation was significantly associated with poor overall and disease-free survival rates (Fig. 7b, c). Moreover, high histopathological grade (Fig. 7d), lymph node metastasis (Fig. 7e) and late TNM stage (Fig. 7f) were also associated with a poor survival rate. Although large tumor size was not significantly different (Fig. 7g), a decrease in the survival rate with increasing tumor size was observed. We also performed univariate and multivariate Cox regression analyses in our research. Both univariate and multivariate Cox regression analyses revealed that patients with high ADAR1 expression, late T category, or lymph node metastasis had poor survival outcomes. Moreover, univariate Cox regression analysis results also showed that patients with a III + IV TNM stage had a poor prognosis (Table 2). Altogether, these data indicated that increased ADAR1 expression may have a significant relationship with poor prognosis in OSCC.

**Table 2:** Cox regression analysis of OSCC survival

Variable	Univariate survival analysis			Multivariate survival analysis		
	HR	95% CI	*P*	HR	95% CI	*P*
Gender (male, female)	0.926	0.464–1.850	0.828	N/A	N/A	N/A
Age (≤60, > 60)	0.746	0.380–1.464	0.394	N/A	N/A	N/A
Smoking (no, yes)	2.11	1.076–4.140	0.03	0.919	0.419–2.015	0.833
T category (T1 + T2, T3 + T4)	5.747	2.673–12.358	< 0.001	8.381	3.453–20.341	< 0.001
N category (N0, N+)	4.8	2.287–10.078	< 0.001	7.405	2.002–27.388	< 0.001
TNM stage (I + II, III + IV)	4.478	1.945–10.312	< 0.001	0.444	0.105–1.873	0.269
Histology grade (1, 2 + 3)	1.746	0.864–3.531	0.121	0.668	0.296–1.509	0.332
ADAR1 expression (Low, High)	6.459	2.666–15.650	< 0.001	5.085	1.936–13.353	0.001

*ADAR1* Adenosine deaminases acting on RNA, *OSCC* Oral squamous cell carcinoma, *HR* Hazard ratio, *95%CI* 95% confidence interval, *P P* value, *T* Tumor size, *N* Lymph node, *TNM* Tumor-node-metastases. Both values signify *P*-value < 0.05

## Discussion

As the sixth most common cancer worldwide, oral squamous cell carcinoma (OSCC) is a grievous health burden [32]. Finding the potential molecular mechanism underlying OSCC invasion and metastasis is urgently needed to improve the prognosis of patients. Recent studies investigating the role of ADAR1 in multiple tumors have shown that ADAR1 works as either an oncogene or a suppressive gene depending on the type of cancer [33]. Upregulation of ADAR1 was detected in some malignant tissues, such as esophageal squamous cell carcinoma [34] and hepatocellular carcinoma [35]. Moreover, brain cancer and metastatic melanoma tissues showed low expression of ADAR1 [36–38]. However, little is known about the effect of ADAR1 on OSCC. In the current study, we provided novel evidence showing that high levels of ADAR1 were detected in both OSCC tissues and cell lines and associated with the clinicopathological characteristics of patients.

ADAR1 has two isoforms: the shorter and constitutive ADAR1 p110 and the full-length interferon-inducible ADAR1 p150 [39]. In human cell lines, it has been proven that ADAR1 p150 is located in the cytoplasm and nucleus, while ADAR1 p110 is mainly expressed in the nucleus [40]. ADAR1 p150 can move between the nucleus and cytoplasm as a shuttling protein. Therefore, ADAR1 p150-mediated A-to-I editing occurs not only in the nucleus but also in the cytoplasm, whereas editing by ADAR1 p110 occurs in the nucleus [41, 42]. Interestingly, shuttling of ADAR1 p110 between the nucleus and cytoplasm was also reported [8]. In the present research, both real-time PCR and western blot results indicated that the ADAR1 p110 was the dominant isoform in OSCC tissues and cell lines. Both nuclear and cytoplasmic expression of ADAR1 p110 were detected in our research. To verify the role of ADAR1 in OSCC, we constructed a lentiviral vector carrying *ADAR1 p110* and knocked down either *ADAR1-p110* or *ADAR1-p150* expression by siRNA transfection. Our data clearly showed that both ADAR1-p110 and ADAR1-p150 could promote the migration, invasion and proliferation of OSCC. However, considering the weaker ADAR1-p150 expression in OSCC tissues and cell lines, we speculate that ADAR1-p110 may play a more important role in OSCC progression.

Recently, heterogeneity among tumor cells is widely accepted. Documented data suggest that a high percentage of surgically resected OSCC patients eventually develop recurrence and distant tumor spread from residual tumors, possibly driven by OSCC-CSCs (oral squamous cell carcinoma-cancer stem cells) [43, 44]. The stemness of OSCC-CSCs is related to tumor initiation and self-renewal properties and further leads to poor prognosis [45, 46]. Many studies suggested that reducing cancer cell stemness could improve radio-sensitivity. By knocking out JARID1B, the cancer stem cell activity was reduced and the stemness radiotherapy sensitivity of OSCC was greatly improved [47]. The presence of CSC phenotype in clonospheres could increase DNA repair capacity [48]. SMC1A knockdown could limit CSC properties and enhance efficacy of radiation therapy [49]. By using western blot assays, we revealed that ADAR1 may play a potential role in promoting the stemness of OSCC cell lines. Overexpression of ADAR1 was conducive to promoting anti-anoikis and proliferation in tumor cells and negatively affecting the prognosis of OSCC. However, radio-resistance assays should be performed in future studies to validate the role of ADAR1.

OSCC cell lines retain epithelium characteristics and lack the ability to rebuild extracellular matrix [50–52]. The acquisition of EMT contributes to cancer cell matrix invasion and metastasis. High expression of VIM and low expression of CDH1 are features of EMT. In our EMT-induction model, the results implied that ADAR1 participated in the EMT process in OSCC cell lines. However, the mechanism of this characteristic remains unknown and needs further study. Nevertheless, the increased expression of six head-neck cancer-related oncogenic miRNAs, which were chosen by reviewing the literature of ADAR1 overexpression, implied that oncogenic miRNAs may be involved in the process. In previous studies, TGF-β was reported to regulate the expression of miRNA at either transcription or post-transcription level [53–55]. The smad4 as a key player in the TGF-β/smad signaling pathway cascades could cause downregulation or upregulation of the miRNAs [56]. Our studies demonstrated that ADAR1 regulated the expression of miRNAs at post-transcription levels while TGF-β regulated ADAR1 expression. With *ADAR1-p110* knockdown, the EMT which induced by TGF-β was reduced, implying ADAR1 play a role in TGF-β induced EMT. However, there still need further experiments to answer whether the TGF-β regulates miRNAs via ADAR1.

MiRNAs could bind to RISC to degrade target mRNA, which may determine the biological characteristics of tumor cells by epigenetic modification [3]. Tumorigenesis and progression involve a series of cellular pathways that control cell biology, and the proteins associated with these cellular pathways may be potential oncogenes. It has been reported that ADAR1 regulates the generation of miRNAs via an editing-independent mechanism [6]. ADAR1 can not only suppress the processing of pri-miRNA to pre-miRNA by interacting with DGCR8 but also increase the processing of miRNAs by forming a heterodimer with Dicer [3, 33, 37]. In our study, we confirmed that ADAR1 could physically interact with Dicer, a key component of the miRNAs processing machinery in the cytoplasm, but weakly or not interact with Drosha, Argonaute1, Argonaute2 or TRBP. Upregulation of six oncogenic miRNAs following the reductions in both pre-miRNA and pri-miRNA by *ADAR1* overexpression and downregulation of these miRNAs by *ADAR1-p110* knockdown implied that ADAR1 may promote OSCC progression by promoting the maturation of oncogenic miRNAs. Furthermore, downregulation of both oncogenic miRNAs and tumor suppressor miRNAs by *Dicer* knockdown implied that ADAR1 may regulate miRNAs expression through Dicer. However, more oncogenic miRNAs and the regulation mechanism should be examined in OSCC cell lines in future research.

## Conclusions

In summary, we demonstrate that upregulation of ADAR1 may promote OSCC progression through facilitating the maturation of oncogenic miRNAs and EMT. Our findings extend the current understanding of the molecular mechanisms of ADAR1 and suggest that ADAR1 may hold promise as a novel therapeutic target in OSCC.

## Additional files

Additional file 1: **Figure S1.** Differences in the expression of *ADAR1-p110* and *ADAR1-p150* between OSCC tissue and matched non-tumor oral mucosa. The expression of *ADAR1-p110* and *ADAR1-p150* were normalized to that of *GAPDH*. The data were expressed as the mean ± SEM (*n* ≥ 3). **p < 0.01. (TIF 1001 kb)

Additional file 2: **Figure S2** Real-time PCR (a) and western blot (b) assays were used to detect ADAR1 expression in Cal27 cells transfected with an *ADAR1* overexpression lentiviral vector. (c) The interference efficiency of three si-*ADAR1-p110* sequences was analyzed by real-time PCR. (d) Western blot assay was used to detect *ADAR1-p110* protein expression in Cal27 and HN4 cells with *ADAR1-p110* knockdown. (e) The interference efficiency of si-*ADAR1-p150* sequences was detected by real-time PCR. (f) Western blot assay was used to detect ADAR1-p150 protein expression in Cal27 and HN4 cells with *ADAR1-p150* knockdown. (g) Representative image indicated the cell cycle of LV-GFP and LV-*ADAR1* groups. (h) Cell proliferation ability was showed in LV-GFP and LV-*ADAR1* groups as detected by CCK-8 assay. (i) Cell proliferation ability was showed in both Cal27 and HN4 cells with *ADAR1-p150* knockdown as detected by CCK-8 assay. The data were summarized for at least three independent experiments. *p < 0.05; **p < 0.01; #*p* > 0.05. (TIF 4844 kb)

Additional file 3: **Figure S3.** Western blot analysis of CDH1 and VIM expression in *ADAR1* overexpressed Cal27 and HN4 cells accompany with *ADAR1-p150* knockdown. (TIF 599 kb)

Additional file 4: **Figure S4.** Real-time PCR assay was used to detect miR-21–3p, miR-18a-3p, miR-210-3p, miR-155-5p, miR-181a-5p and miR-19a-3p expression. The data were summarized from at least three independent experiments. *p < 0.05; **p < 0.01; #*p* > 0.05. (TIF 902 kb)

Additional file 5: **Table S1.** The primary antibodies used in western blot. (XLSX 34 kb)

Additional file 6: **Table S2.** Clinicopathological profiles of 61 primary OSCC patients. OSCC, oral squamous cell carcinoma; P, P value; T, tumor size; N, lymph node; M, distant metastasis; TNM, tumor-node-metastases. (XLSX 26 kb)

## Data Availability

The datasets used and/or analyzed during the current study are available from the corresponding author on reasonable request.

## Abbreviations

ADARs: Adenosine deaminases acting on RNA
CDH1: Cadherin 1
dsRNA: Double-stranded RNA
EMT: Epithelial-mesenchymal transition
HNSCC: Head and neck squamous cell carcinoma
HOK: Human oral keratinocyte
miRNA: MicroRNA
OSCC: Oral squamous cell carcinoma
OSCC-CSC: Oral squamous cell carcinoma-cancer stem cell
PCNA: Proliferating cell nuclear antigen
POU5F1: POU class 5 homeobox1
pre-miRNA: Precursor transcript of miRNA gene
pri-miRNA: Primary transcript of miRNA gene
SOX2: SRY-box 2
TGF: Transforming growth factor
VIM: Vimentin
