# Supplementary figures and images for: ADAR1 promotes the epithelial-to-mesenchymal transition and stem-like cell phenotype of oral cancer by facilitating oncogenic microRNA maturation
Source: J Exp Clin Cancer Res. 2019 Jul 17;38:315. doi: 10.1186/s13046-019-1300-2 (PMC6637647; doi:10.1186/s13046-019-1300-2)

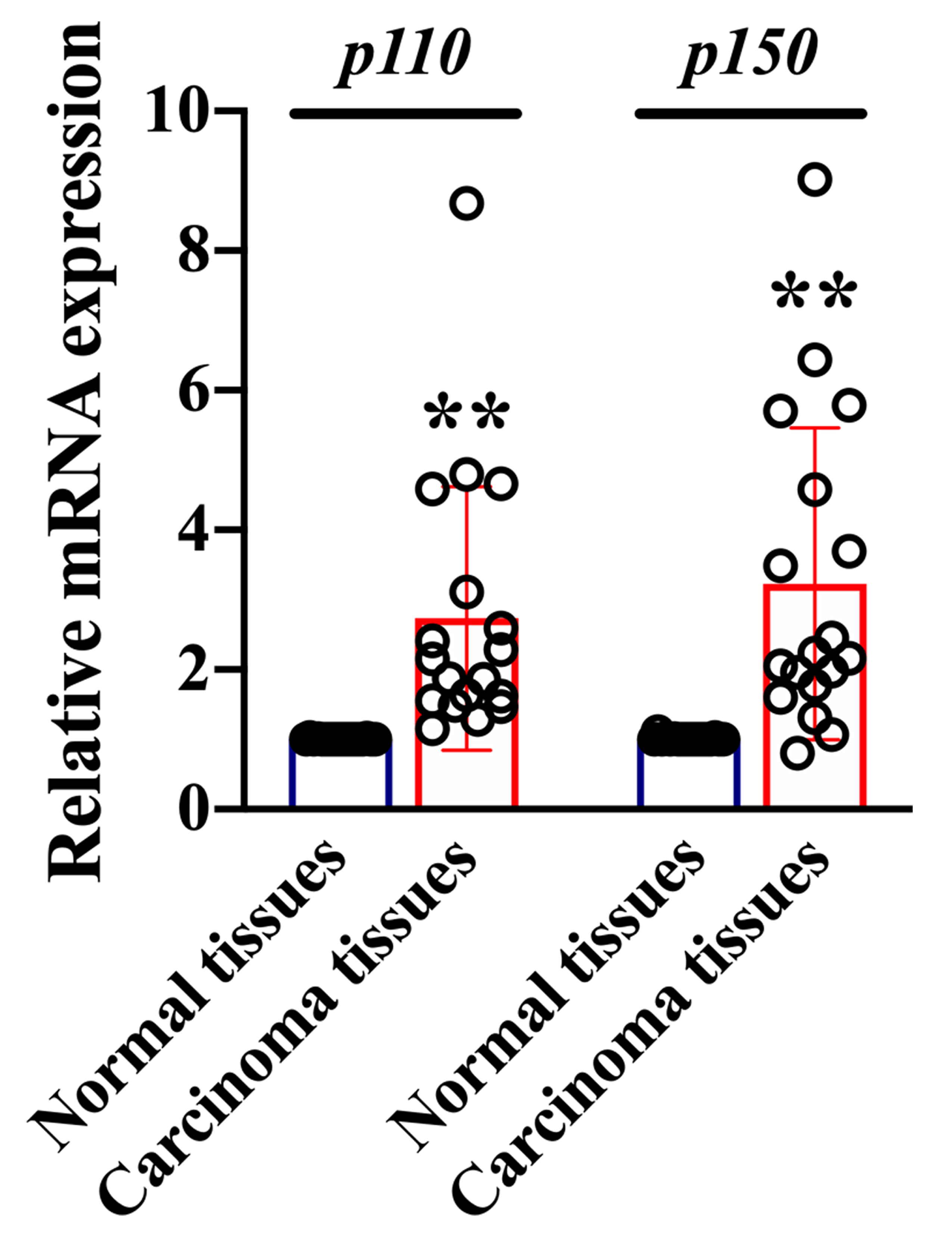

Supplement: Supplementary file 1 — Figure S1. Differences in the expression of ADAR1-p110 and ADAR1-p150 between OSCC tissue and matched non-tumor oral mucosa. The expression of ADAR1-p110 and ADAR1-p150 were normalized to that of GAPDH. The data were expressed as the mean ± SEM (n ≥ 3). **p < 0.01. (TIF 1001 kb) [file 13046_2019_1300_MOESM1_ESM.tif]

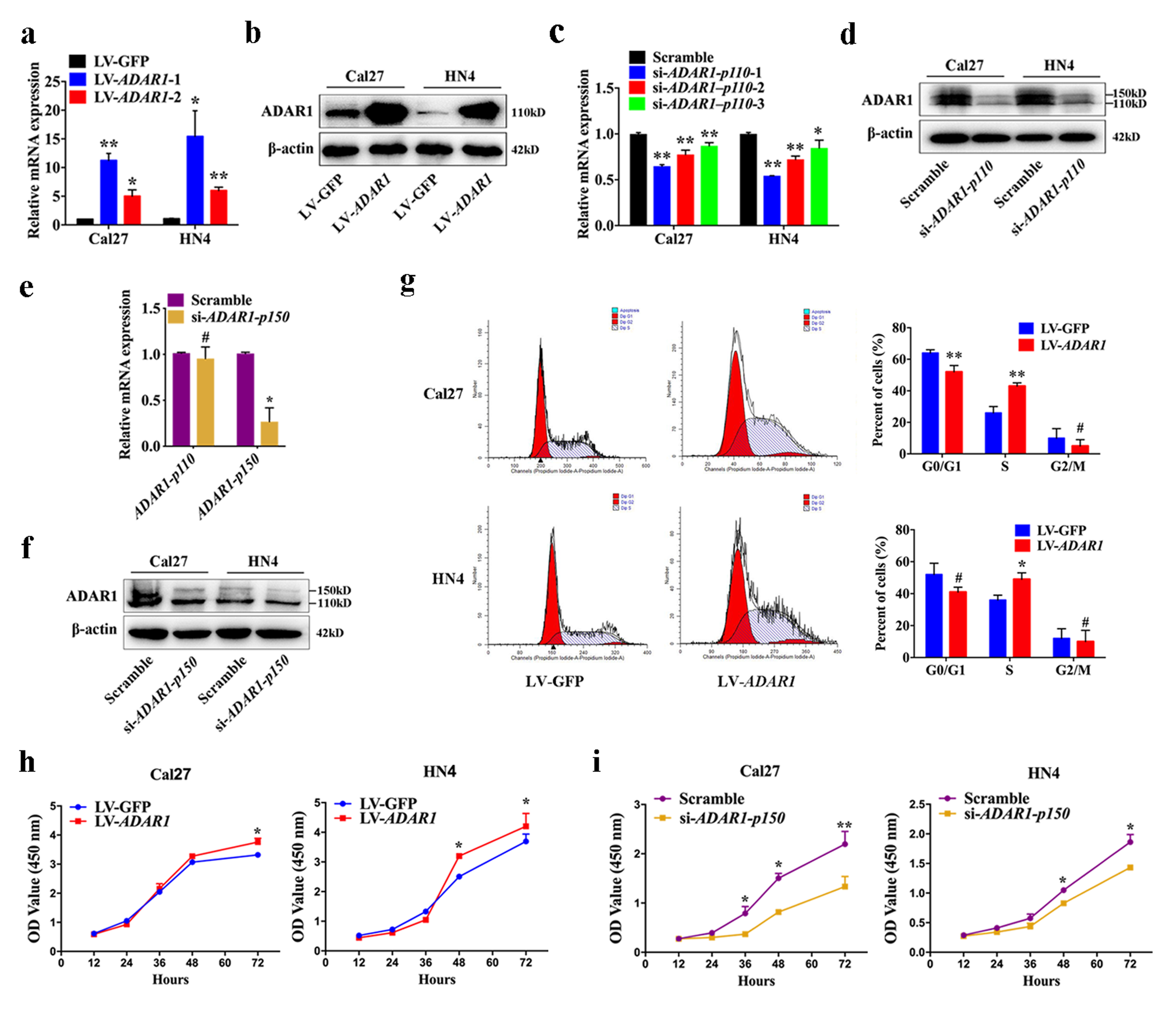

Supplement: Supplementary file 2 — Figure S2 Real-time PCR (a) and western blot (b) assays were used to detect ADAR1 expression in Cal27 cells transfected with an ADAR1 overexpression lentiviral vector. (c) The interference efficiency of three si-ADAR1-p110 sequences was analyzed by real-time PCR. (d) Western blot assay was used to detect ADAR1-p110 protein expression in Cal27 and HN4 cells with ADAR1-p110 knockdown. (e) The interference efficiency of si-ADAR1-p150 sequences was detected by real-time PCR. (f) Western blot assay was used to detect ADAR1-p150 protein expression in Cal27 and HN4 cells with ADAR1-p150 knockdown. (g) Representative image indicated the cell cycle of LV-GFP and LV-ADAR1 groups. (h) Cell proliferation ability was showed in LV-GFP and LV-ADAR1 groups as detected by CCK-8 assay. (i) Cell proliferation ability was showed in both Cal27 and HN4 cells with ADAR1-p150 knockdown as detected by CCK-8 assay. The data were summarized for at least three independent experiments. *p < 0.05; **p < 0.01; #p > 0.05. (TIF 4844 kb) [file 13046_2019_1300_MOESM2_ESM.tif]

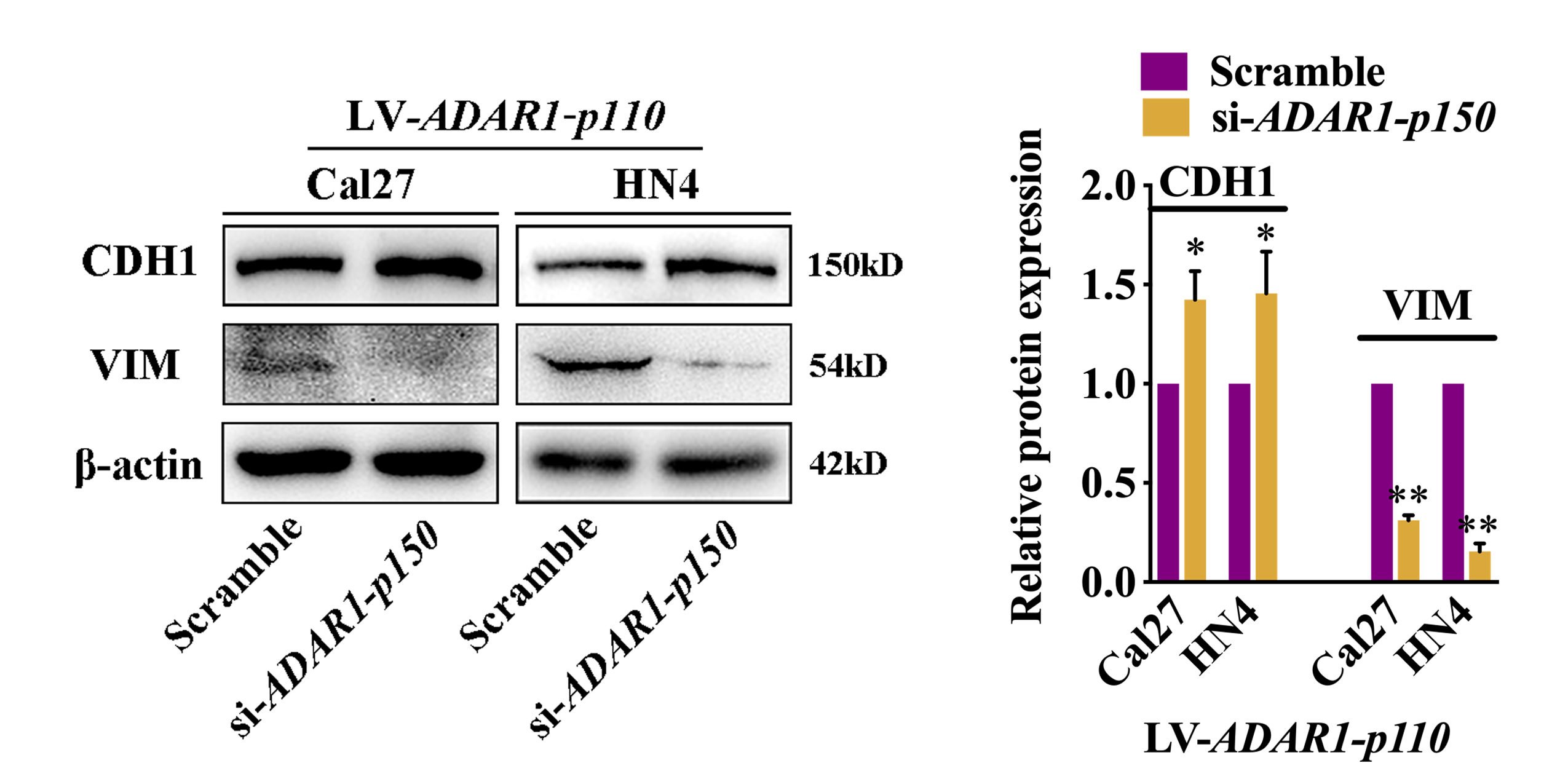

Supplement: Supplementary file 3 — Figure S3. Western blot analysis of CDH1 and VIM expression in ADAR1 overexpressed Cal27 and HN4 cells accompany with ADAR1-p150 knockdown. (TIF 599 kb) [file 13046_2019_1300_MOESM3_ESM.tif]

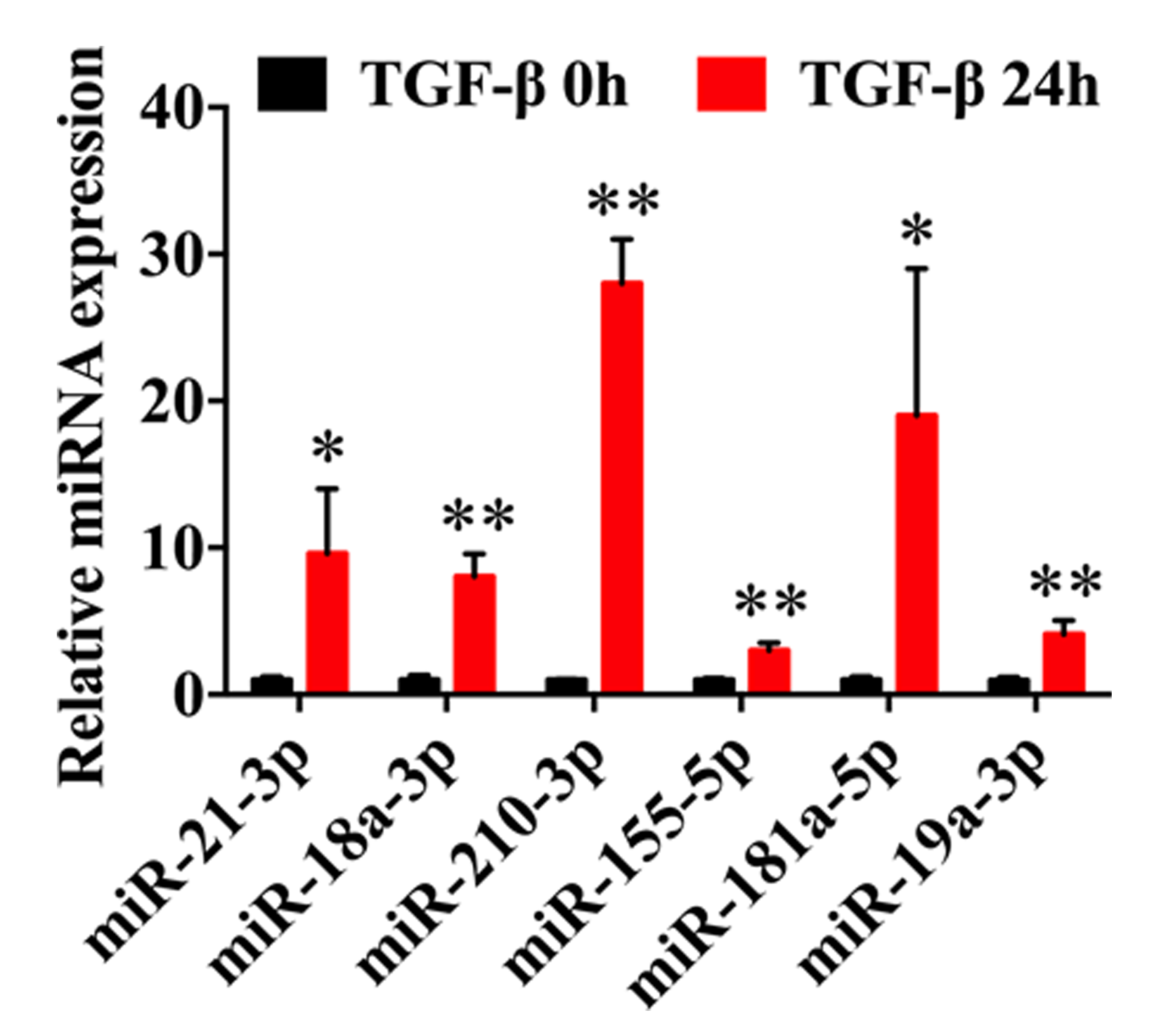

Supplement: Supplementary file 4 — Figure S4. Real-time PCR assay was used to detect miR-21–3p, miR-18a-3p, miR-210-3p, miR-155-5p, miR-181a-5p and miR-19a-3p expression. The data were summarized from at least three independent experiments. *p < 0.05; **p < 0.01; #p > 0.05. (TIF 902 kb) [file 13046_2019_1300_MOESM4_ESM.tif]
